# P-621. The association between pediatric vaccine-limiting and non-completion of the combined 7-vaccine series among US military families, 2010–2019

**DOI:** 10.1093/ofid/ofae631.819

**Published:** 2025-01-29

**Authors:** Monica Burrell, Celeste Romano, Anna Bukowinski, Clinton Hall, Gia Gumbs, Ava Marie Conlin, Nanda Ramchandar

**Affiliations:** Naval Health Research Center, San Diego, California; Naval Health Research Center, San Diego, California; Naval Health Research Center, San Diego, California; Naval Health Research Center, San Diego, California; Naval Health Research Center, San Diego, California; Naval Health Research Center, San Diego, California; Naval Medical Center San Diego, San Diego, California

## Abstract

**Background:**

Recent work reported gaps in completion of the pediatric combined 7-vaccine series among US military families. To determine the role of vaccine hesitancy as a contributing factor to undervaccination, we examined the prevalence of vaccine-limiting patterns and measured the association between these patterns and vaccine non-completion.

**Methods:**

Department of Defense Birth and Infant Health Research program data were used to identify children born at military facilities, 2010–2019. Immunizations were identified through age 2 years using vaccine administered and Current Procedural Terminology codes from military facility immunization records and health care encounter records, respectively. Consistent vaccine limiters received < 3 antigens at each vaccine visit (ie, all limited visits). Episodic vaccine limiters had ≥1 limited vaccine visit and only 3 visits with ≥3 antigens. Non-limiters had no limited visits or ≥4 visits with ≥3 antigens. Modified Poisson regression models estimated associations between vaccine-limiting patterns and non-completion of the combined 7-vaccine series by age 2 years.

**Results:**

Overall, 271,510 children were identified for analysis. The prevalence of consistent vaccine-limiting was 0.3% (n=758); episodic vaccine-limiting was 13.6% (n=36,879). Each group was disproportionately represented among the population non-complete for the 7-vaccine series (1.1% and 27.5%, respectively). Consistent vaccine limiters had 5.6 (95% confidence interval [CI]: 5.5-5.7) times the risk and episodic vaccine limiters had 3.0 (95% CI: 2.9-3.0) times the risk of non-completion compared with non-limiters.

**Conclusion:**

Although few US military families consistently limited pediatric vaccinations, episodic vaccine-limiting throughout the first 2 years of life was common, and both vaccine-limiting patterns were associated with higher risk for vaccine series non-completion. Findings suggest greater attention to vaccine-limiting is needed in this population.

**Disclosures:**

**All Authors**: No reported disclosures

